# microRNAs Shape Myeloid Cell-Mediated Resistance to Cancer Immunotherapy

**DOI:** 10.3389/fimmu.2020.01214

**Published:** 2020-07-22

**Authors:** Elena Daveri, Elisabetta Vergani, Eriomina Shahaj, Laura Bergamaschi, Stefano La Magra, Michela Dosi, Chiara Castelli, Monica Rodolfo, Licia Rivoltini, Viviana Vallacchi, Veronica Huber

**Affiliations:** Unit of Immunotherapy of Human Tumors, Department of Research, Fondazione IRCCS Istituto Nazionale dei Tumori, Milan, Italy

**Keywords:** microRNAs, myeloid-derived suppressor cells, immunotherapy, immune checkpoints, therapy resistance, extracellular vesicles

## Abstract

Immunotherapy with immune checkpoint inhibitors can achieve long-term tumor control in subsets of patients. However, its effect can be blunted by myeloid-induced resistance mechanisms. Myeloid cells are highly plastic and physiologically devoted to wound healing and to immune homeostasis maintenance. In cancer, their physiological activities can be modulated, leading to an expansion of pro-inflammatory and immunosuppressive cells, the myeloid-derived suppressor cells (MDSCs), with detrimental consequences. The involvement of MDSCs in tumor development and progression has been widely investigated and MDSC-induced immunosuppression is acknowledged as a mechanism hindering effective immune checkpoint blockade. Small non-coding RNA molecules, the microRNAs (miRs), contribute to myeloid cell regulation at different levels, comprising metabolism and function, as well as their skewing to a MDSC phenotype. miR expression can be indirectly induced by cancer-derived factors or through direct miR import via extracellular vesicles. Due to their structural stability and their presence in body fluids miRs represent promising predictive biomarkers of resistance, as we recently found by investigating plasma samples of melanoma patients undergoing immune checkpoint blockade. Dissection of the miR-driven involved mechanisms would pave the way for the identification of new druggable targets. Here, we discuss the role of these miRs in shaping myeloid resistance to immunotherapy with a special focus on immunosuppression and immune escape.

## Introduction

Myeloid cells are involved in inflammatory processes, including cancer, and their accrual to the tumor microenvironment (TME) leads to immunosuppression and angiogenesis, thereby promoting tumor growth. Thanks to their plasticity, they are acknowledged cancer allies, negative prognostic factors, and pharmacological targets. Low/negative HLA-DR expression (1) defines monocytic myeloid-derived suppressor cells (CD14^+^HLA-DR^low/neg^; M-MDSCs), which influence cancer aggressiveness and resistance to immune checkpoint inhibitors (ICIs) (2). We focused on myeloid cells for more than a decade and first defined M-MDSCs in melanoma patients (3). We dissected underlying mechanisms via an *in vitro* tumor extracellular vesicle (EV)-healthy donor monocyte-MDSC model and identified a set of causally involved microRNAs (miRs), the “MDSC-miRs.” miRs are small non-coding RNAs of ~22 nucleotides, which modulate biological processes by mostly interacting with the 3′-untranslated region (UTR) of the target messenger RNA (mRNA). An imperfect base-pair interaction induces translational repression, while a perfectly base-paired miR directly cleaves the mRNA (4, 5). However, some miRs can also bind the 5′-UTR of mRNA, upregulating its translation (6). We measured increased MDSC-miR levels in circulating CD14^+^ cells and lesions of melanoma patients in association with myeloid infiltrates and peripheral blood MDSC accrual (7, 8). Matching of MDSC-miR predicted target genes with EV-MDSC transcriptional profile revealed miR involvement in chemotaxis, adhesion, and differentiation of myeloid cells. The upregulation of MDSC-miRs, including miR-146a, miR-146b, miR-155, miR-125b, miR-100, let-7e, miR-125a, and miR-99b, in baseline plasma predicted resistance to ICIs (8). *In vitro*, MDSC-miR antagonists relieved the suppressive potential of patients' monocytes leading to autologous T cell reactivation. Thus, MDSC-miRs could account for myeloid deregulation, implying an involvement of blood factors in the epigenetic control of MDSC functions. A higher MDSC frequency is associated with poor prognosis, even upon immunotherapy, anticipating a reduced treatment efficacy. Pharmacological MDSC reduction, inhibition of their suppressive activities or promotion of their differentiation are under testing at preclinical and clinical levels (9, 10). The functional roles of miR expression by immune cells remain controversial. In case of MDSC-miRs, their overexpression impacts myeloid cell differentiation and polarization by participating in immunosuppressive pathways. Like other miRs, also MDSC-miRs are detectable in EVs, whose size correlates with M-MDSC frequency (8). Tumor and immune cell EVs attracted interest as reservoirs of functional messages exchanged between adjacent cells in the TME and at a distance. EV membrane guarantees content integrity, enabling safe traveling of proteins, lipids, and genetic material to interaction-prone cells. Major efforts are dedicated to investigate EVs as biomarkers of response or drivers of resistance mechanisms to ICIs.

This review discusses the role of MDSC-miRs in shaping myeloid resistance to immunotherapy with a focus on immunosuppression and escape.

### The Role of miRs in Cancer Therapy Resistance

As oncomiRs or tumor-suppressors, miRs can promote or inhibit cancer development. They directly target cell proliferation and apoptosis genes, thus being involved in chemotherapy resistance, drug target deregulation, and drug metabolism mechanisms (11). In immunotherapy, miRs can control the success of ICIs by targeting PD-1 and PD-L1, MHC-antigen presentation machinery, and TLR signaling (12). Among MDSC-miRs, miR-155 suppresses PD-L1 through directly binding the 3′-UTR of PD-L1 in human lymphatic endothelial cells (13). The reduction of PD-L1 expression and the consequent disruption of the PD-L1/PD-1 axis may contribute to sustaining T cell antitumor responses, thereby synergizing with ICIs to improve cancer immunotherapy outcome. This miR contributes essentially to mounting of CD8^+^ T cell responses by restraining T cell senescence and exhaustion through epigenetic silencing of transcription factors determining their terminal differentiation (14). Moreover, miR-155 expression correlates with TCR stimulation of tumor-infiltrating T cells in melanoma patients (15). The MDSC-miR-146a, 146b, 155, and let-7, bind to the 3′-UTR of TLRs or TLR-associated genes resulting in post-translational TLR signaling repression and inflammatory response modulation (16). Similarly, the MDSC-miR-125a and let-7e regulate the inflammatory response and the IL-10-mediated tolerance to LPS, by targeting the TLR4 pathway in monocytes (17). TLR4 can promote expansion of PD-L1^+^ MDSCs, an effect mediated via HSP86-TLR4 signaling pathway activation (18). Since specific miRs can directly activate TLRs expressed at endosomal level (19, 20), MDSC-miRs might target these proteins and contribute to expanding PD-L1 expressing MDSCs. Thus, targeting MDSC-miRs might potentiate ICI-based immunotherapy.

The development of therapeutic antagomiRs and miR mimics have entered phase I and II clinical studies (21). The DNA-single strand antagomiRs are usually designed on first-generation antisense oligonucleotides or modified with locked nucleic acids to reduce the oncomiR activity by competition with the native cancer-suppressing target transcripts (22). MiR mimics are double strand oligonucleotides that enter the native cellular process mimicking pre-miR duplex (23). Cobomarsen (MRG-106), a miR-155 inhibitor, has entered phase I trials to study safety and potential efficacy following local or intravenous administration in lymphoma and leukemia patients (24).

Despite the therapeutic potential of miRs, their delivery remains challenging, due to undesired off-target effects, hindered cell uptake, and short circulation half-life (25). Synthetic nanoparticles (NPs) mediate specific cell uptake and prevent miR clearance (26). In preclinical models, effective miR supply was obtained via neutral lipid emulsion-based approach for miRs of the let-7 family, as well as neutral liposomes and synthetic polyethylenimine-based nanocarriers for miR-145. Lastly, pH low insertion peptide-modified antagomiRs were able to inhibit the oncomiR miR-155 (27). Otherwise, miR-155-loaded NPs can repolarize tumor-associated macrophages (TAMs) from pro-tumorigenic M2 to anti-inflammatory M1-like phenotype, reversing the immunosuppressive TME (28). In clinical setting, NP-based miR manipulation comprises liposomal (DOPC)-encapsulated siRNAs targeting EphA2 in solid tumors (29), bacterial derived nanocells EDVs (EnGeneIC Delivery Vehicle), or TargomiRs, for miR-16 mimic delivery (30). EVs may be also suitable for miR delivery (31). Healthy donor plasma miR-loaded EVs promoted apoptosis in HCC cells (31), while miR-sponge engineered EVs reduced glioblastoma volume in rats (32). Finally, the natural exchange of endogenous miRs between immune cells, such as miR-155 and miR-146a carried by dendritic cell EVs, controls inflammatory gene expression or promotes apoptotic cell clearance, as in case of endothelial cell EVs transferring miR-125a to macrophages (33).

Specific miR inhibition is accomplished by Small Molecule Inhibitors of miRs (SMIRs), which target synergistically tumor cells and oncomiRs, such as linifanib. This VEGF- and PDGF-receptor tyrosine kinase-inhibitor effectively inhibits the oncogenic function of miR-10b in preclinical cancer models (34). Finally, several miRs are related to tumor radioresistance management, where, thanks to the inhibition of ATM protein, they can modulate DNA damage response sensitizing tumor cells to radiotherapy (35).

### Epigenetic Regulation of Immune Cell Functions by MDSC-miRs

The upregulation of miR-146a, miR-146b, miR-155, miR-125b, miR-100, let-7e, miR-125a, and miR-99b can skew immune cells into inhibitors of response to immune and other cancer therapies (Table 1). Of note, five miRs out of eight show a coordinated expression pattern due to their transcription as clusters. The miR-125a~99b~let-7e cluster is hosted in the first intron of the long non-coding RNA NCRNA00085, whereas miR-125b, miR-100, and let-7a are hosted in MIR100HG (17, 58). A clear association of miR-125a~99b~let-7e cluster and acquisition of a myeloid immunosuppressive phenotype has been demonstrated (17, 36, 59, 60). In particular, stimulation of monocytes with GM-CSF, IL-4, and R848 TLR7/8 agonist upregulates the miR-125a~99b~let-7e cluster, activates STAT3, and induces the acquisition of an immunosuppressive phenotype. Conversely, the depletion of the cluster reverses immunosuppressive functions and MDSC phenotype hallmarks, by downregulating PD-L1 and IDO, while increasing HLA-DR expression. This contributes to STAT3 stabilization through downregulation of TRIB2, a suppressor of MAPK signaling, and SOCS1, a key regulator of cytokine signaling and STAT3 inhibitor. The miR-125a~99b~let-7e cluster is negatively regulated by IFNγ, while it is induced by STAT3 and SMAD3, in turn activated by IL-10 and TGFβ immunoregulatory cytokines. miR-125a and let-7e also exert their anti-inflammatory activity by targeting the TLR signaling pathway molecules TLR4, CD14, and IRAK1, leading to decreased pro-inflammatory cytokine release by myeloid cells (17, 59).

**Table 1:** Role of MDSC-miRs in tumorigenesis and response to cancer therapies.

**miR**	**Cells**	**Expression**	**Target genes/Pathways**	**Phenotype**	**References**
miR-125a~ 99b~let-7e	Monocytes	**↑**	TRIB; SOCS1	Immunosuppressive properties mediated by STAT3 activation	(36)
miR-125a and let-7e	Monocytes	**↑**	TLR4; CD14; IRAK1	**↓** Anti-inflammatory activity and cyto/chemokines	(17)
miR-125b	Macrophages	**↑**	IRF4	Acquisition of M1 phenotype	(37)
	T cells	**↑**	IFNG; IL10RA; IL2RB; PRDM1	Suppression of CD4^+^ T cell differentiation	(37)
	T cells	**↑**	CD107a; TNFA; IFNG	Inhibition of γδ T cell activation	(37)
miR-100	Tregs	**↑**	SMAD2	**↓** Treg differentiation and **↑** plasticity	(38)
miR-146b	Macrophages	**↑**	IRF5	**↓** M1 macrophage and inflammation	(39)
miR-146a	Monocytes	**↓**	TRAF6; IRAK1	**↑** chronic NFkB driving myeloid malignancy	(40, 41)
	Breast cancer	**↓**	TRAF6/IRAK1	**↑** NFkB activity and metastasis	(42)
	Endometrial cancer	**↑**	NIFK-AS1	**↑** M2-like phenotype of TAMs	(43)
	Hepatocellular carcinoma	**↑**	STAT3	Immunosuppression by ↑TGFβ, IL17, VEGF and ↓type I IFN	(44)
	Melanoma	**↑**	STAT1/IFNγ axis; PD-L1	Melanoma migration, MDSC promotion and resistance to ICIs	(8, 45)
	MDSCs	**↑**	NFkB	**↓** NFkB-mediated inflammation	(46)
	T cells	**↑**	IFNγ and perforin	**↓** ICI-mediated irAEs severity	(47)
miR-155	Breast cancer	**↑**	SOCS1/SHIP1	Activation of STAT3 signaling and pro-tumor inflammation	(48)
	Myeloid cells	**↓**	C/EBP-β	Breast tumor growth by MDSC infiltration and TAM tolerance	(49, 50)
	MDSCs	**↓**	HIF-1α	**↑** MDSC recruitment and function, **↑** solid tumor growth	(51)
	MDSCs	**↑**	SHIP1	**↑** STAT3 activation and expansion of functional MDSCs	(52)
	Colorectal cancer	**↑**	SOCS1	**↑** MDSC activity and tumor growth	(53)
	T cells	**↑**	SHIP1	**↑** IFNγ production, **↑** T cell-mediated antitumor immunity	(54)
	Melanoma	**↑**	ND	MDSC induction **↑** resistance to immunotherapy	(8)
	T cells	**↑**	T cell activation markers	**↑** T cell response	(55)
	T cells	**↑**	PRC2/Phf19	**↑** cancer immunotherapy by **↑** CD8^+^ T cell function	(14)
	T cells	**↑**	TIM3	Cytolytic activity of CD8^+^ T cells against HCC	(56)
	T cells	**↑**	ND	**↑** antitumor activity of CD8^+^ T cells	(57)

*ND, not defined; ↑, increased; ↓, decreased*.

MIR100HG and its encoded miR-125b and miR-100 are induced by TGFβ, the main cytokine released by M2 macrophages (61). TGFβ promotes cancer epithelial-to-mesenchymal transition (EMT) through MIR100HG induction and SMAD2/3 transcription factor activation. The dysregulation of this cluster is also causally linked with drug resistance in several tumor types (58, 62). In immune cells, miR-125b expression is usually linked to antitumor M1-like macrophages, whereas in T cells it inhibits CD4 T cell differentiation and γδ T cell activation (37). In contrast, little is known about miR-100 expression and function in immune cells. In regulatory T cells (Tregs) increased levels of the edited variant of miR-100 changes its target gene from MTOR to SMAD2, resulting in limited differentiation and increase of Treg plasticity (38).

### MDSC-miRs and Response to Immunotherapy

Under physiological conditions the miR-146 family (miR-146a and miR-146b) and miR-155 actively control innate immunity, whereas in cancer these miRs have gained attention for their deregulation and acquisition of oncogenic roles. Both are transcriptionally regulated by NFkB, but with opposite functions: miR-146 represents the anti-inflammatory and miR-155 the pro-inflammatory counterpart. miR-146a/b act as negative feedback regulators of TLR signaling through inhibition of the NFkB pathway by downregulation of TRAF6 and IRAK1 (63), thereby dampening the production of pro-inflammatory mediators (64). On the other hand, miR-146b is also induced by TLR4 signaling via an IL-10-mediated STAT3-dependent loop (65), and it inhibits macrophage activation by targeting IRF5 (39). miR-146a is an essential regulator of immune cell activation and malignant transformation (64), and knockout mice are affected by chronic NFkB dysregulation and myeloid malignancies (40, 41). Several studies proposed miR-146a as an immunotherapeutic target: its overexpression reduces the metastatic potential of breast cancer (BC) cell lines through NFkB inhibition (42), whereas it supports the M2-like phenotype of TAMs in endometrial cancer (43). In a preclinical model of HCC, miR-146a inhibition alters the STAT3 activation-associated cytokine profile improving the anti-tumor effect of lymphocytes (44). Mastroianni et al. identified miR-146a as a central negative regulator of the STAT1/IFNγ axis, affecting migration, proliferation, and inducing PD-L1 expression. Combined PD-1 blockade and miR-146a antagomiR improve survival of melanoma-bearing mice (45). We found that high miR-146a levels, concomitantly with the other MDSC-miRs, are associated with MDSC induction and ICI resistance (8). In myeloid leukemia, miR-146a mimics can inhibit tumorigenic NFkB activity (46). Finally, miR-146a is also involved in ICI-mediated immune-related adverse events (irAEs), as shown by knockout mice exhibiting increased T cell activity and inflammation during ICI intake. These effects could be restrained by miR-146a mimics (47).

The pro-inflammatory miR-155 is induced upon TLR/IFNγ stimulation in monocyte/macrophages and drives their response by regulating mRNA targets with inhibitory effects on innate immune cell activation (66). In tumor cells, intrinsic miR-155 mediates pro- or anti-tumor effects (67). Similarly to miR-146a, miR-155 upregulation promotes cell proliferation, colony formation, and xenograft tumor growth in BC models by negative regulation of SOCS1 and SHIP1, leading to constitutive STAT3 activation and pro-tumor inflammation (48). Deficiency of miR-155 can also foster tumor growth through MDSC recruitment and potentiation of their tumor promoting functions, as demonstrated in BC. Here, miR-155 loss in myeloid cells impairs TAM activation, while in tumor cells it stimulates C/EBP-β-mediated cytokine production in turn stimulating tumor-infiltrating MDSCs (49, 50). Similar results were obtained in mouse models of melanoma and lung cancer (51). As for other miRs also miR-155 appears to cover apparently contradictory roles depending on the expressing cell or the setting. Li et al. showed that upregulated miR-155 together with miR-21 led to MDSC expansion, whereas their loss reversed this effect. In particular, by targeting SHIP1 and PTEN these miRs synergistically increase STAT3 activity, promoting MDSCs (52). In this line, loss of miR-155 can enhance antitumor T cell activity by reducing MDSC immunosuppression and tumor infiltration (53). In contrast, miR-155 expression by T cells promotes antitumor immunity and ICIs hinder miR-155-deficiency-induced immune escape (54). We found that miR-146a and miR-155 along with the other MDSC-miRs contribute to MDSC induction (8), suggesting that the expression levels of different miRs can influence the fine-tuning of pro- or anti-inflammatory pathways depending on the cell type. Interestingly, in tumors with high mutational burden, such as melanoma and lung cancer, miR-155 was associated with a strong immune signature and improved clinical outcomes (55). Likewise, miR-155 potentiates immunotherapy through epigenetic regulation of CD8^+^ T cell differentiation via PRC2/Phf19 signaling (14). Yan and coworkers demonstrated that miR-155-induced downregulation of TIM3, a negative immune checkpoint, enhanced the cytolytic activity of anti-HCC CD8^+^ T cells (56). Finally, miR-155 overexpression can optimize CD8^+^ T cell antitumor activity and improve adoptive-transfer in low-affinity antigen tumors (57).

### EVs as miR Shuttles and MDSC Modulators

All cell types release EVs, membrane-surrounded structures devoted to intercellular communication. EVs are present in body fluids including plasma, serum, lymph, urine, saliva, tears, and milk (68). Their content of proteins, nucleic acids, lipids, and their stability, make EVs potential biomarkers and therapeutic targets of disease (69, 70). Recent evidence shows the ability of tumor-derived EVs to blunt anti-tumor immunity at multiple levels (71). They can operate within the TME or at a distance by boosting angiogenesis, triggering tumor cell EMT, activating cancer-associated fibroblasts, and shaping the immune environment toward a condition of immune escape. Tumor EVs can induce myeloid cell dysfunctions and increase MDSC expansion (7, 8, 18, 72, 73). Indeed, EVs derived from melanoma cell cultures as well as those from plasma of melanoma patients contain the MDSC-miRs and promote the acquisition of MDSC characteristics by healthy donors' monocytes (8). The potential of these miRs to induce such dramatic changes might depend on their integrity, protected by the EV membrane, as well as on their way of transfer. In fact, the internalization of whole EVs carrying different miRs or EV receptor-ligand interaction might account for diverse effects (74). In support of our findings, Gerloff et al. (75) found that miR-125b encapsulated in melanoma EVs promotes a TAM phenotype in macrophages through targeting of lysosomal acid lipase (LIPA). In fact, LIPA deficiency stimulates MDSC expansion in mice, and their tumor promoting functions are driven by mTOR pathway overactivation (76).

As a major obstacle to immunotherapy, it is crucial to study MDSC effects in the TME (77). Myeloid EVs may support immune activation or tolerance (78). Like other cells, also MDSCs release EVs, taking part in intercellular communication. Proteomics of MDSC EVs of BALB/c mice bearing 4T1 or 4T1-IL-1β^+^ mammary carcinoma showed a higher expression of 63 pro-inflammatory proteins in 4T1-IL-1β^+^ mice, due to a more inflammatory environment. The MDSC chemotactics S100A8 and S100A9 are abundant in MDSC EVs and polarize macrophages into M2 phenotype (79). These pro-inflammatory proteins are characterized by multiple ubiquitination sites, and MDSC EVs were identified as carriers of enzymes catalyzing ubiquitination (80). Interestingly, EVs from TME-resident MDSC display a stronger immunosuppressive potential than those deriving from spleen or bone marrow MDSCs, suggesting the existence of different phenotypes and functions (81). In contrast, the miR content of MDSC EVs is still elusive, but the dissection could contribute to targeting MDSC EVs release and spreading (82). EVs modulate innate and adaptive immune responses via ligand-receptor interaction or via miRs (83, 84). Indeed, a set of miRs including miR-155, regulate PD-L1 protein expression (85) and induce MDSCs if released via EVs by CLL cells (86). EVs also express immune checkpoints. Actually, PD-L1 carried by EVs was investigated for its role as biomarker and functional inducer of PD-1-mediated immunosuppression (87–90). TIM3 and GAL9 bound to EVs were proposed as prognostic biomarkers in NSCLC patients (91).

### Translational Implications

Large scale profiling studies demonstrated the association of specific circulating miRs with certain types of human cancer, proposing miRs as biomarkers (92). Their detection could contribute to early cancer diagnosis, patient stratification, and evaluation of therapy outcomes (93). miRs can be found free or EV-bound in peripheral blood or other body fluids (94). Among MDSC-miRs, the miR-125a~99b~let-7e cluster was identified as a potential diagnostic biomarker in many tumor types. Colorectal and ovarian cancer patients display lower levels of EV-bound miR-99b compared to healthy controls (95, 96). Dysregulated levels of free circulating let-7e were observed in retinoblastoma, papillary thyroid carcinoma, lung, and prostate cancer (97–100), whereas altered levels of EV-bound let-7 characterize esophageal adenocarcinoma and lung cancer patients (101, 102). miR-125a also represents a potential biomarker of treatment outcome for HCC patients (103) and altered levels of this miR were detected in certain blood malignancies (104, 105), where they predicted response to chemotherapy, as demonstrated in patients with myelodysplastic syndromes (106). In serum, increased miR-146b levels correlate with papillary thyroid carcinoma recurrence (92), while elevated miR-146a is associated with higher overall response rate and survival in NSCLC (107). Furthermore, lower EV-bound miR-146a levels correlate with cisplatin resistance and shorter progression-free survival in NSCLC patients (108). BC patients display high plasma miR-155 levels and in the absence of disease, its increase is associated with treatment failure (109). Interestingly, also urinary miR-155 can be correlated with BC development (93, 110). In NSCLC, an increase of miR-155 in plasma identifies stage I-II patients, implying this miR as diagnostic tool (93, 111), although it was not suitable as a prognostic biomarker (112). miR-155 expression is also related to risk of relapse in colorectal cancer patients and chemoresistance in pancreatic ductal adenocarcinoma, where anti-apoptotic mechanisms are driven by tumor cell exchange of miR-155 containing EVs (93, 113, 114). Concerning MIR100HG, reduced miR-100 expression coincides with diagnosis and prognosis of bladder cancer (115). In contrast, higher circulating miR-100 levels were found in HCC and esophageal squamous cell carcinoma patients and predicted poor survival (116, 117). Lastly, circulating miR-125b was identified as a biomarker of diagnosis and poor prognosis in NSCLC, BC, colorectal, and epithelial ovarian cancer patients, also during chemotherapy or after surgery (118–123).

## Conclusion

**Figure 1:**
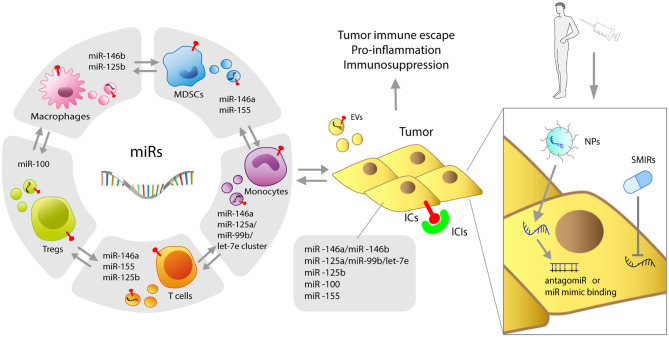
Graphic representation of miRs shaping myeloid resistance to ICIs. Immune and tumor cells, both expressing miRs, mutually interact through miR-loaded EVs. MDSC-derived miRs, including miR-146a, miR-146b, miR-155, miR-125b, miR-100, let-7e, miR-125a, and miR-99b can intervene in cancer progression and interfere with the success of cancer immunotherapy by regulating immune checkpoints (ICs) and different molecular immune targets. Delivery of antagomiRs or miR mimics with NPs, as well as SMIR drugs, represents the current therapeutic strategies to overcome resistance to ICIs induced by miRs. NPs, nanoparticles; SMIR, small molecule inhibitor of miR; ICIs, immune checkpoint inhibitors; EVs, extracellular vesicles.

Despite major advances, the role of miRs, including MDSC-miRs, expressed by immune cells remains controversial. For instance, both pro and antitumoral potentials are ascribed to miR-155, depending on its expression levels (124). Of interest is also their interplay: miR-146a^−/−^ mice succumb to chronic inflammation and miR-155 expressed by T cells contributes to shortening lifespan by activating autoimmunity (125). The continuous technical improvement will facilitate in-depth investigations of the finely-tuned mechanisms governing the miR balance, expression levels, and consequent repression/overexpression of target genes to clarify the mechanisms governing myeloid cell dysfunctions and MDSC activity. This will be of major relevance also for cancer therapies. In fact, similarly to SMIRs, also ICIs may induce changes in myeloid MDSC-miR expression potentially related to clinical responses. The complex tumor-immune relationship regulated by miRs and the miR-based therapeutic approaches are summarized in Figure 1. Thus, the dissection of therapy-induced miR modulation in immune cells may contribute to decipher and antagonize resistance mechanisms.

## Author Contributions

All authors wrote and revised the manuscript. LB made the figure. All authors have read and agreed to the submitted version of the manuscript.

## Conflict of Interest

The authors declare that the research was conducted in the absence of any commercial or financial relationships that could be construed as a potential conflict of interest.
